# Nutritional status and mental health burden among adults living with disabilities in urban Ghana: a cross-sectional study

**DOI:** 10.1186/s12889-026-28025-5

**Published:** 2026-06-05

**Authors:** Jonathan Annan-Asare, Castroline Appiah-Kubi, Astrid Naa Adei Ashie

**Affiliations:** 1 Department of Nutrition and Dietetics, School of Allied Health Sciences, University of Health and Allied Sciences, Ho, PMB 31 Ghana; 2 Department of Biochemistry and Biotechnology, College of Science, Kwame Nkrumah University of Science and Technology, Kumasi, Ghana

**Keywords:** Disability, Malnutrition, Depression, Anxiety, Urban health, Ghana, Public health

## Abstract

**Background:**

Adults living with disabilities experience disproportionate health challenges, including higher rates of malnutrition, poverty, and limited healthcare access. However, evidence on their nutritional and mental health status in low- and middle-income countries remains limited. This study assessed the burden of malnutrition, depression, and anxiety among adults living with disabilities in the Accra Metropolis, Ghana’s capital.

**Methods:**

A community-based cross-sectional study was conducted between May and July 2024 among 350 adults with physical, visual, hearing, or intellectual disabilities recruited using stratified convenience sampling. The sample deliberately exceeded the calculated minimum (*n* = 104) to improve precision and representation. Nutritional status was assessed using anthropometric measurements following WHO protocols. Dietary intake was evaluated using two non-consecutive 24-hour recalls (multiple-pass method) and a food frequency questionnaire. Depression and anxiety symptoms were assessed using the validated screening tools – the PHQ-9 and GAD-7. Descriptive statistics were used to summarize participant characteristics and outcome measures.

**Results:**

A double burden of malnutrition was observed, with 28.9% (*n* = 101) underweight and 31.4% (*n* = 110) overweight or obese. Moderate-to-severe depressive symptoms were present in 22.9% of participants, and 55.4% reported moderate-to-severe anxiety. No statistically significant associations were observed between selected micronutrient intakes and anxiety or depression symptom scores.

**Conclusions:**

Adults living with disabilities in urban Ghana experience a substantial double burden of malnutrition, reflecting a double burden of both undernutrition (28.9%) and overnutrition (31.4%), with nearly a quarter experiencing moderate-to-severe depression (22.9%) and over half reporting anxiety (55.4%). These findings underscore the need for disability-inclusive public-health interventions that integrate nutrition and mental health services within urban health systems.

## Introduction

Disability is a major and growing global health and development concern. The World Health Organization estimates that approximately 15% (over 1 billion people) of the world’s population lives with some form of disability, with higher absolute burdens in low- and middle-income countries (LMICs) where health systems and social protection are commonly weaker [1]. People living with disabilities (PLWD) experience intersecting risks for poor health outcomes driven by poverty, social exclusion, and limited access to health and social services [2, 3]. In Ghana, recent census data indicate that disability affects a sizeable minority of the population and that prevalence varies by type and by urban–rural location [4]. Despite legal frameworks for disability rights, PLWD continue to experience barriers to healthcare, education, and employment that increase vulnerability to poor nutrition and mental health problems [5, 6].

Malnutrition and disability are bidirectionally related: malnutrition may contribute to disability (e.g., developmental delays), while disability may increase malnutrition risk (e.g., feeding difficulties or reduced mobility) [7–9]. The global nutrition landscape has also shifted: many LMIC urban settings now experience the “double burden” of malnutrition — defined as the coexistence of undernutrition and overweight/obesity— within the same communities and even within households [10, 11]. Ghana is no exception — systematic reviews and national data document rising urban overweight and obesity together with persistent undernutrition in vulnerable groups [12, 13]. Yet few studies disaggregate these patterns for adults living with disabilities; available evidence tends to focus on children with disabilities or on the general population, leaving a critical knowledge gap for adult disability populations in urban African settings [7, 14].

Mental disorders — particularly depression and anxiety — are leading contributors to global disability, and are closely associated with social deprivation, chronic physical illness, and reduced functioning [15, 16]. PLWD are at elevated risk of psychological distress for multiple reasons: stigma, unemployment, reduced social participation, pain, and inadequate access to mental health services [17–19]. Recent scoping and systematic reviews report substantially higher prevalence of depression among people with disabilities compared with the general population, with prevalence estimates frequently far above national averages, often 2–3 times higher than the general population estimates in LMIC studies [20, 21]. In Ghana and other parts of sub-Saharan Africa, mental health services are limited, and routine screening for depression and anxiety is not widely available in community disability services [22, 23].

There is also growing interest in the bidirectional relationship between nutrition and mental health. Observational studies and recent randomized trials suggest that overall diet quality and specific nutrients may influence mood and risk of depressive symptoms; however, evidence is heterogeneous, and causality cannot be assumed from cross-sectional data [24–26]. Adults living with disabilities may face multiple nutritional risks (reduced dietary diversity, food insecurity, and feeding difficulties) that could plausibly contribute to psychological distress or modify the relationship between diet and mental health [7, 27]. Yet empirical studies on dietary adequacy and mental health, specifically among PLWD in LMIC urban contexts, are scarce.

Given these substantial evidence gaps, particularly the lack of data on nutritional status and mental health among adults with disabilities in urban African settings, this study aimed to: (1) estimate the prevalence of undernutrition and overnutrition among adults living with disabilities in the Accra Metropolis; (2) determine the prevalence and severity of depressive and anxiety symptoms; (3) assess dietary intake patterns and micronutrient adequacy; and (4) examine associations between micronutrient intakes and mental health symptoms. By combining anthropometry and dietary assessment with validated screening tools (PHQ-9 and GAD-7), our goal was to provide multi-dimensional evidence to inform disability-inclusive nutrition and mental health programming in urban Ghana.

## Methods

### Study design and setting

A community-based cross-sectional study was conducted between May and July 2024 in the Accra Metropolis, Ghana. Accra, the administrative and economic capital of the country, is a highly urbanized setting with substantial socioeconomic heterogeneity and a diverse population. The metropolitan context provides a relevant urban low- and middle-income setting for examining nutritional status and mental health outcomes among adults living with disabilities. Participants were recruited from disability organizations, community centers, and healthcare facilities.

### Study population

Adults aged 18–40 years living with physical, visual, hearing, or intellectual disabilities were eligible. Exclusion criteria included participants who were acutely ill, requiring hospitalization, or immediate medical attention, as well as those who did not provide informed consent.

### Sample size determination

Using an estimated disability prevalence of 6.5% for urban Ghana, a 95% confidence level, and a 5% margin of error, the minimum required sample size was calculated as 94 participants. Allowing for a 10% non-response rate, a final sample size of approximately 105 participants was considered adequate for the study.

The sample size was calculated using the single-population proportion formula:

$$n_{o}=\left[Z^{2}\times\,p\left(1-p\right)\right]/\left(d^{2}\right)$$

Where:

- *Z* = 1.96 (95% confidence level)
- *p* = estimated disability prevalence (6.5% = 0.065) (urban disability prevalence from Ghana 2021 PHC [4])
- *d* = margin of error (0.05)

$$n_{o}=\left[\left(1.96\right)^2\,\times\,0.065\left(1-0.065\right)\right]/\left(0.05\right)^2\,=93.44$$

Adjusting for a 10% non-response rate:

- n_adjusted = 93.4/ 0.9*≈*104

The study deliberately oversampled (350 vs. 104 calculated) to: (1) improve precision of estimates, (2) ensure adequate representation across disability types, and (3) account for anticipated measurement challenges. This approach enhanced the robustness and interpretability of estimates derived from anthropometric and mental health assessments and is consistent with methodological recommendations for research involving vulnerable and hard-to-reach populations [1, 6, 27].

### Sampling technique

Participants were recruited using stratified convenience sampling: disability types (physical, visual, hearing, and intellectual) were used as strata, with convenience sampling within each stratum due to the absence of a population-based registry of adults with disabilities in Accra. Participants were recruited through disability organizations, community centers, and healthcare facilities. Target recruitment aimed for approximately equal representation across disability types.

### Ethical considerations

Ethical approval was obtained from the University of Health and Allied Sciences Research Ethics Committee, UHAS-REC A.7 [78] 24–25. Prior to data collection, the purpose, procedures, potential benefits, and minimal risks of the study were explained to all participants in English, Twi, or Ga, as preferred by the participant. Written informed consent or thumbprint for participants unable to write was obtained from each participant before enrolment. For participants with intellectual disabilities, consent was obtained from legal guardians, with participant assent where possible. Participation was entirely voluntary, and participants were informed of their right to decline or withdraw from the study at any time without consequences. Strict measures were taken to ensure privacy, anonymity, and confidentiality. Personal identifiers were collected only for consent forms, which were stored separately from study data, and all data were securely stored with access limited to the research team and used solely for research purposes. Paper records were stored in locked cabinets; electronic data was password-protected and stored on encrypted devices. The study posed minimal risk to participants. Potential risks included fatigue from lengthy interviews and emotional distress from mental health questions. Participants were offered breaks, and referral information for mental health services was provided. Participants received nutritional counseling and referrals for follow-up care where indicated. No monetary compensation was provided. The study was conducted in accordance with established ethical principles for research involving human participants.

### Data collection procedures and measures

Data were collected by trained research assistants. Interviews were conducted in English or local languages (Twi, Ga) as preferred by participants. Each assessment session lasted approximately 45–60 min.

#### Anthropometric assessment

Weight and height were measured following WHO standardized protocols [28]. Weight was measured using a calibrated digital scale (± 0.1 kg accuracy), and height using a stadiometer (± 0.1 cm accuracy). Measurements were taken with participants in light clothing and without shoes. All measurements were taken in duplicate, and the average was used for analysis. Body mass index (BMI) was calculated and classified using WHO BMI cut-offs: underweight (< 18.5 kg/m²), normal (18.5–24.9 kg/m²), overweight (25.0–29.9 kg/m²), and obese (≥ 30.0 kg/m²). Waist-to-hip ratio was calculated after measuring waist and hip circumferences with measuring tapes. Waist circumference was measured at the narrowest point between the lower rib and iliac crest, and hip circumference at the widest point over the buttocks. For participants unable to stand, alternative methods were used: recumbent length for height and segmental measurements, where appropriate.

#### Dietary assessment

Dietary intake was assessed using two non-consecutive 24-hour dietary recalls (3–7 days apart) with the multiple-pass method to enhance recall accuracy and a semi-quantitative food frequency questionnaire (FFQ) to assess usual intake over the past month. The FFQ was adapted from previously validated food frequency questionnaires commonly used in nutritional epidemiology and modified to reflect locally consumed foods in the study setting. The instrument was pilot tested among a small group of participants to assess clarity, relevance, and cultural appropriateness before the main data collection. Portion sizes were estimated using standardized household measures, food models, and food photographs. Dietary data were analyzed using NutriSurvey software in combination with the Ghana Food Composition Tables to estimate energy and nutrient intakes.

#### Mental health assessment

Depressive symptoms were assessed using the Patient Health Questionnaire-9 (PHQ-9), and anxiety symptoms using the Generalized Anxiety Disorder-7 questionnaire (GAD-7), both validated screening tools [29, 30]. PHQ-9 scores: 0–4 minimal, 5–9 mild, 10–14 moderate, 15–19 moderately severe, 20–27 severe depression. GAD-7 scores: 0–4 minimal, 5–9 mild, 10–14 moderate, 15–21 severe anxiety. Both tools have been validated in Ghanaian populations [23, 31]. Questionnaires were administered through face-to-face interviews by trained research assistants. For participants with visual impairments, questions were read aloud; for those with hearing impairments, sign language interpreters were used where necessary.

### Statistical analysis

Data were analyzed using IBM SPSS Statistics version 25 (IBM Corp., Armonk, NY, USA). Descriptive statistics were used to summarize participants’ sociodemographic characteristics. Continuous variables were reported as means and standard deviations, while categorical variables were presented as frequencies and percentages. Pearson correlation analysis was conducted to assess the linear associations between micronutrient intakes and symptoms of anxiety and depression among adults living with disabilities in Accra. Correlation coefficients (r) were calculated, and statistical significance was determined at a two-tailed significance level of p-value < 0.05. Normality of continuous variables was assessed using Shapiro-Wilk tests and visual inspection of Q-Q plots. Complete case analysis was used; participants with missing data for specific variables were excluded from relevant analyses. Assumptions of linearity and homoscedasticity were checked prior to correlation analysis. Correlation coefficients were interpreted as: 0.00-0.09 (negligible), 0.10–0.29 (weak), 0.30–0.49 (moderate), ≥ 0.50 (strong). Given the exploratory nature of the correlation analyses, no adjustment for multiple comparisons was applied; results should be interpreted with caution.

## Results

### Socio-demographic characteristics of study participants

The study included 350 adults living with disabilities, with representation across physical, visual, hearing, and intellectual disability categories. The mean age was 29.5 years ± 6.8, indicating a relatively young disability population. The majority were Christians (72.9%). Educational attainment varied, with the largest group having junior high school education (25.4%), while a substantial proportion (20.9%) had no formal education. In terms of employment, about 31.2% were students, while 27.4% were unemployed. Regarding disability type, 34.9% had physical disabilities, 32.0% visual impairments, and 33.1% multiple disabilities (hearing and speech impairments). Most disabilities were acquired (58.9%), with a mean onset age of 10.7 ± 5.3 years, while 41.1% were congenital. Detailed socio-demographic characteristics of the participants are presented in Table 1.

**Table 1:** Socio-demographic characteristics of participants

Variables	Mean ± SD	Frequencies (*n* = 350)	Percentages (%)	95% CI
Gender				
Male		179	51.1	45.9–56.3
Female		171	48.9	43.6–54.1
Age (years)	29.5 ± 6.8			
18–24		93	26.6	22.2–31.5
25–29		80	22.9	18.7–27.6
30–34		80	22.9	18.7–27.6
35–40		97	27.6	23.3–32.7
Age of disability onset (years)	10.7 ± 5.3			
Marital status				
Single		158	45.1	39.8–50.4
Married		86	24.6	20.3–29.3
Divorced		52	14.9	11.5–18.9
Widowed		54	15.4	12.0-19.6
Religion				
Christianity		255	72.9	68.2–77.5
Islam		95	27.1	22.5–31.8
Education				
Tertiary		44	12.6	9.5–16.6
Senior High School		78	22.2	18.2–26.9
Junior High School		89	25.4	21.1–30.2
Primary		66	18.9	15.1–23.3
No Formal Education		73	20.9	16.9–25.5
Employment Status				
Employed		75	21.4	17.4–26.1
Self-employed		70	20.0	16.1–24.5
Unemployed		96	27.4	22.9–32.4
Student (not employed)		109	31.2	26.5–36.2
Type of Disability				
Physical		122	34.9	29.9–40.1
Visual		112	32.0	27.1–37.2
Multiple		116	33.1	28.2–38.3
Onset of Disability				
Congenital		144	41.1	35.9–46.3
Acquired		206	58.9	53.7–64.0

Multiple = Hearing and speech impairments

*CI* Confidence Interval

### Participant’s anthropometric characteristics and nutritional status distribution (*N* = 350)

Table 2 summarizes the anthropometric characteristics and nutritional status of the study participants. The mean body weight was 58.6 ± 2.1 kg, with an average height of 161.3 ± 2.6 cm, resulting in a mean body mass index (BMI) of 22.5 ± 4.7 kg/m². Mean waist circumference and hip circumference were 79.2 ± 7.1 cm and 87.8 ± 8.4 cm, respectively, with a mean waist-to-hip ratio of 0.9 ± 0.02, indicating elevated abdominal obesity risk (WHO cut-off: <0.90 men, < 0.80 women). Based on the WHO BMI classification, 28.9% (*n* = 101) of participants were underweight, 39.7% (*n* = 139) had a normal BMI, 22.0% (*n* = 77) were overweight, and 9.4% (*n* = 33) were obese. This pattern reflects the coexistence of undernutrition and overnutrition within the same population.

**Table 2:** Anthropometric measurements and nutritional status among adults living with disabilities in Accra

Variable	Mean ± SD	Frequency (*n*)	Percentage (%)
Weight (kg)	58.6 ± 2.1		
Height (cm)	161.3 ± 2.6		
Hip Circumference (cm)	87.8 ± 8.4		
BMI (kg/m^2^)	22.5 ± 4.7		
Underweight		101	28.9
Normal weight		139	39.7
Overweight		77	22.0
Obese		33	9.4
WHR	0.9 ± 0.02		
Normal		229	65.4
High		121	34.6
Males	0.9 ± 0.02		
Normal		169	94.4
High		10	5.6
Females	0.9 ± 0.03		
Normal		60	35.1
High		111	64.9
Waist Circumference	79.2 ± 7.1		
Normal		286	81.7
High		64	18.3
Males	78.8 ± 7.1		
Normal		172	96.1
High		7	3.9
Females	79.6 ± 7.0		
Normal		114	66.7
High		57	33.3
Body fat (%)	20.8 ± 5.1		
Normal		304	86.9
High		46	13.1
Males	20.7 ± 4.9		
Normal		164	91.6
High		15	8.4
Females	21.0 ± 5.2		
Normal		140	81.8
High		31	18.1
Visceral Fat	7.2 ± 2.2		
Normal		271	77.4
High		79	22.6
Males	7.1 ± 2.2		
Normal		134	74.9
High		45	25.1
Females	7.4 ± 2.1		
Normal		137	80.1
High		34	19.9

Data presented as mean *±* standard deviation, and as frequencies, *n* (%)

*Abbreviations*: *BMI* Body Mass Index, *WHR* Waist-to-Hip Ratio, *WC* Waist Circumference

BMI classification based on WHO criteria: Underweight (<18.5 kg/m²), Healthy weight (18.5–24.9 kg/m²), Overweight (25.0–29.9 kg/m²), Obese (≥30.0 kg/m²)

WHR classification based on WHO criteria: High WHR defined as >0.90 for males and >0.85 for females

Visceral fat classification based on WHO criteria for both males & females: ≤ 9%: Normal, >9: High Body fat classification based on WHO criteria: for females: < 25: Normal, ≥ 25: High, for males: < 30: Normal, ≥ 30 high

WC classification based on WHO criteria: High WC defined as ≥ 9 cm for males and ≥ 80cm for females

### Dietary intake patterns of participants

Table 3 summarizes the frequency of food consumption among adults living with disabilities in the Accra Metropolis. Rice emerged as the most frequently consumed staple, with the majority of participants, 230 (65.7%), reporting daily intake. Other starchy foods, including yam, plantain, gari, waakye, and fufu, were consumed mainly two to five times per week. Akple (a traditional Ghanaian staple prepared from fermented maize (corn) and cassava dough cooked in boiling water to form a firm, smooth, dough-like consistency. It is a carbohydrate-dense food) showed comparatively lower consumption frequency.

**Table 3:** Frequency of consumption of food groups among adults living with disabilities in Accra

Food Type	Daily		2–3/Week		4–5/Week		2–3/Month		Once/Month		Never	
	*n*	%	*n*	%	*n*	%	*n*	%	*n*	%	*n*	%
Starches												
Rice	230	65.7%	30	8.6%	30	8.6%	60	17.1%	0	0%	0	0%
Yam	44	12.6%	106	30.3%	90	25.7%	34	9.7%	47	13.4%	29	8.3%
Plantain	52	14.9%	87	24.9%	99	28.3%	32	9.1%	55	15.7%	25	7.1%
Akple	32	9.1%	46	13.1%	63	18%	66	18.9%	82	23.4%	61	17.4%
Gari	44	12.6%	74	21.1%	114	32.6%	26	7.4%	64	18.3%	29	8.3%
Waakye	34	9.7%	79	22.6%	109	31.1%	36	10.3%	57	16.3%	35	10%
Fufu	43	12.3%	79	22.6%	103	29.4%	37	10.6%	60	17.1%	28	8%
Meat												
Chicken	32	9.1%	93	26.6%	108	30.9%	28	8%	61	17.4%	28	8%
Beef	40	11.4%	80	22.9%	105	30%	34	9.7%	68	19.4%	23	6.6%
Goat Meat	31	8.9%	77	22%	105	30%	47	13.4%	64	18.3%	26	7.4%
Gizzard	26	7.4%	89	25.4%	118	33.7%	25	7.1%	60	17.1%	32	9.1%
Sausage	30	8.6%	87	24.9%	102	29.1%	32	9.1%	58	16.6%	41	11.7%
Fishes												
Salmon	29	8.3%	92	26.3%	99	28.3%	44	12.6%	58	16.6%	28	8%
Herrings	30	8.6%	161	46%	50	14.3%	38	10.9%	41	11.7%	30	8.6%
Mackerel	29	8.3%	103	29.4%	94	26.9%	37	10.6%	64	18.3%	21	6%
Smoked fish	32	9.1%	80	22.9%	108	30.9%	28	8%	64	18.3%	38	10.9%
Eggs												
Eggs	22	6.3%	212	60.6%	28	8%	28	8%	22	6.3%	38	10.9%
Cereals												
Bread	27	7.7%	28	8%	206	58.9%	32	9.1%	21	6%	36	10.3%
Noodles	51	14.6%	89	25.4%	92	26.3%	2	0.6%	59	16.9%	32	9.1%
Pizza	39	11.1%	70	20%	100	28.6%	41	11.7%	75	21.4%	25	7.1%
Oats	27	7.7%	45	12.9%	79	22.6%	80	22.9%	84	24%	35	10%
Wheats	30	8.6%	36	10.3%	79	22.6%	55	15.7%	119	34%	34	9.7%
Dairy Products												
Milk/Milk product	27	7.7%	24	6.9%	32	9.1%	30	8.6%	204	58.3%	28	8%
Yoghurt	44	12.6%	94	26.9%	88	25.1%	32	9.1%	66	18.9%	29	8.3%
Fat and Oils												
Margarine/ butter	33	9.4%	23	6.6%	29	8.3%	34	9.7%	207	59.1%	24	6.9%
Mayonnaise	57	16.3%	74	21.1%	134	38.3%	25	7.1%	29	8.3%	31	8.9%
Vegetable oil	81	23.1%	78	22.3%	75	21.4%	49	14%	62	17.7%	5	1.4%
Sunflower oil	39	11.1%	95	27.1%	107	30.6%	30	8.6%	46	13.1%	33	9.4%
Vegetables												
Cabbage/ Lettuce	37	10.6%	23	6.6%	27	7.7%	28	8%	198	56.6%	37	10.6%
Tomato	63	18%	104	29.7%	89	25.4%	30	8.6%	36	10.3%	28	8%
Green pepper	32	9.1%	36	10.3%	34	9.7%	35	10%	185	52.9%	28	8%
Carrot	22	6.3%	90	25.7%	76	21.7%	22	6.3%	34	9.7%	95	27.1%
Onion	42	12%	78	22.3%	115	32.9%	56	16%	33	9.4%	26	7.4%
Fruits												
Orange	23	6.6%	31	8.9%	30	8.6%	203	58%	37	10.6%	26	7.4%
Pineapple	43	12.3%	82	23.4%	81	23.1%	47	13.4%	66	18.9%	31	8.9%
Apple	40	11.4%	62	17.7%	60	17.1%	64	18.3%	84	24%	40	11.4%
Banana	29	8.3%	52	14.9%	73	20.9%	70	20%	88	25.1%	38	10.9%
Watermelon	44	12.6%	86	24.6%	89	25.4%	36	10.3%	59	16.9%	36	10.3%
Mango	38	10.9%	74	21.1%	78	22.3%	54	15.4%	74	21.1%	32	9.1%
Pawpaw	34	9.7%	57	16.3%	75	21.4%	83	23.7%	64	18.3%	37	10.6%
Sweets												
Sugar	29	8.3%	204	58.3%	32	9.1%	32	9.1%	26	7.4%	27	7.7%
Chocolate	22	6.3%	39	11.1%	57	16.3%	92	26.3%	100	28.6%	57	16.3%
Cookies/Cake	46	13.1%	92	26.3%	84	24%	47	13.4%	65	18.6%	16	4.6%
Pastries	30	8.6%	65	18.6%	95	27.1%	44	12.6%	80	22.9%	44	12.6%
Beverages												
Packed Fruit Juice	30	8.6%	29	8.3%	23	6.6%	212	60.6%	29	8.3%	27	7.7%
Soft Drinks	54	15.4%	64	18.3%	92	26.3%	44	12.6%	57	16.3%	39	11.1%
Energy Drinks	40	11.4%	75	21.4%	115	32.9%	37	10.6%	59	16.9%	24	6.9%
Coffee	52	14.9%	71	20.3%	99	28.3%	32	9.1%	61	17.4%	35	10%
Tea	34	9.7%	62	17.7%	85	24.3%	57	16.3%	81	23.1%	31	8.9%
Local Drinks	31	8.9%	75	21.4%	105	30%	47	13.4%	64	18.3%	28	8%

Animal-source protein intake was moderate overall. Chicken, beef, goat meat, gizzard, and sausage were most commonly consumed two to three times per week. Fish constituted a more regular protein source than meat, with herrings showing the highest frequency of intake. Egg consumption was relatively frequent, whereas intake of milk and milk products was low, with a substantial proportion of participants reporting rare or no consumption.

Cereal products such as bread and noodles were widely consumed, particularly bread, which was commonly eaten four to five times per week. In contrast, intake of whole grains such as oats and wheat products was less frequent. Vegetable oil was the most commonly used fat, followed by sunflower oil, while margarine, butter, and mayonnaise were consumed less frequently.

Vegetable consumption was dominated by tomatoes, onions, and green peppers, whereas leafy vegetables and carrots were consumed less often. Fruit intake was generally low, with most fruits consumed occasionally or monthly rather than daily. Sugar and sweetened products were commonly consumed, with sugar intake reported several times per week by a large proportion of participants. Soft drinks, energy drinks, and local beverages were consumed with moderate frequency.

### Energy and nutrient intake of participants

Table 4 presents the mean nutrient intakes, nutrient adequacy ratios (NAR), and prevalence of adequacy among adults living with disabilities in Accra. Overall, dietary intake patterns revealed suboptimal micronutrient adequacy despite moderate macronutrient intake.

**Table 4:** Nutrient adequacy ratio and mean adequacy ratio among adults living with disabilities in Accra

Nutrients Intake	Mean Intake ± SD	Mean NAR	Adequate (%)	Inadequate (%)
Macronutrients				
Carbohydrates (g)	250.8 ± 23.8	-	-	-
Protein (g)	51.0 ± 4.3	0.9	283 (80.9)	67 (19.1)
Fats (g)	52.7 ± 6.3	-	-	-
Energy (kcal)	1906.1 ± 53.2	0.7	223 (63.7)	127 (36.3)
Micronutrients				
Calcium (mg)	1250.3 ± 18.5	0.7	195 (55.7)	155 (44.3)
Iron (mg)	11.3 ± 1.4	0.4	45 (12.9)	305 (87.1)
Zinc (mg)	7.1 ± 0.7	0.5	64 (18.2)	286 (81.7)
Vitamin A (µ_RAE)	59.7 ± 5.2	0.2	34 (9.7)	316 (90.3)
Vitamin C (mg)	79.3 ± 8.9	1.3	289 (82.6)	61 (17.4)
Thiamin (mg)	1.1 ± 0.4	0.8	218 (62.3)	132 (37.7)
Riboflavin (mg)	0.9 ± 0.2	0.6	56 (16.0)	294 (84.0)
Niacin (mg)	16.3 ± 1.7	0.9	243 (69.4)	107 (30.6)
Vitamin B6 (mg)	1.6 ± 0.1	0.9	281 (80.3)	69 (19.7)
Folate (µg)	309.3 ± 11.3	0.6	91 (26.0)	259 (74.0)
Vitamin B12 (mg)	5.7 ± 1.2	2.1	280 (80.0)	70 (20.0)
Selenium (µg)	45.8 ± 5.6	0.5	116 (33.1)	234 (66.9)
Vitamin D (µg)	9.7 ± 0.3	0.6	111 (31.7)	239 (68.3)

**Mean MAR ± SD**	**Inadequate (%)**	**Moderately Adequate (%)**		**Adequate (%)**
0.65 ± 0.1	82 (23.4)	153 (43.7)		115 (32.9)

^*^kcal kilocalories, *g* gram, *mg* milligram, *µg* microgram, *RAE* Retinol Activity Equivalent, *NAR* Nutrient Adequacy Ratio, *adequate* = ≥ 0.66 *inadequate *= < 0.66, *SD* Standard Deviation, *MAR *Mean Adequacy Ratio, *adequate* = ≥ 0.7, *moderately adequate* = 0.55–0.69, *inadequate* = < 0.55

Mean daily energy intake was 1906.1 ± 53.2 kcal, with a corresponding mean NAR of 0.7, indicating borderline adequacy; however, 36.3% of participants had inadequate energy intake. Protein intake was relatively sufficient (51.0 ± 4.3 g; NAR = 0.9), with over 80% of participants meeting adequacy requirements.

In contrast, several micronutrients demonstrated marked inadequacies. Iron intake was particularly low (NAR = 0.4), with 87.1% of participants classified as inadequate. Similarly, zinc (NAR = 0.5; 81.7% inadequate) and riboflavin (NAR = 0.6; 84.0% inadequate) showed substantial deficits. Vitamin A intake was critically low (NAR = 0.2), with over 90% of participants failing to meet adequacy thresholds.

Calcium intake was moderate (NAR = 0.7), though 44.3% remained inadequate. Folate (NAR = 0.6; 74.0% inadequate) and vitamin D (NAR = 0.6; 68.3% inadequate) also showed considerable insufficiency, while selenium intake was inadequate in 66.9% of participants.

Conversely, some nutrients demonstrated relatively good adequacy. Vitamin C intake exceeded requirements (NAR = 1.3; 82.6% adequate), and vitamin B12 showed high adequacy (NAR = 2.1; 80.0% adequate). Niacin and vitamin B6 also exhibited generally adequate intake levels among the majority of participants.

The overall dietary quality, as reflected by the Mean Adequacy Ratio (MAR), was 0.65 ± 0.1, indicating moderate adequacy at the population level. However, only 32.9% of participants achieved adequate dietary quality, while 23.4% were classified as inadequate, and the largest proportion (43.7%) fell within the moderately adequate range.

### Mental health status of study participants

Table 5 summarizes the prevalence of anxiety and depression among adults living with disabilities in Accra. Among the 350 participants, 15.1% reported minimal anxiety and 29.4% mild anxiety, while 51.1% and 4.3% were classified as having moderate and severe anxiety, respectively. Overall, 55.4% met the threshold for anxiety based on combined moderate to severe categories.

**Table 5:** Prevalence of anxiety and depressive symptoms among participants

Variable	Mean ± SD	Frequency (*n*)	Percentage (%)
Anxiety	13.1 ± 1.2		
Minimal (0–4)		53	15.1
Mild (5–9)		103	29.4
Moderate (10–14)		179	51.1
Severe (15–21)		15	4.3
Total classified as anxious (Moderate-to-severe anxiety)		194	55.4
Depression	8.5 ± 1.7		
Minimal (0–4)		88	25.1
Mild (5–9)		182	52.0
Moderate (10–14)		63	18.0
Moderately severe (15–19)		13	3.8
Severe (20–27)		4	1.1
Total classified as depressed (Moderate-to-severe depression)		80	22.9

For depressive symptoms, 25.1% of participants reported minimal depression, and 52.0% mild depression. Moderate, moderately severe, and severe depression were reported by 18.0%, 3.8% and 1.1% of participants, respectively, resulting in an overall depression prevalence of 22.9%.

The earlier prevalence figures were generated using a preliminary analysis and wrong categorization, in which mild, moderate, moderately severe, and severe symptom categories were inadvertently combined when estimating the overall prevalence of depression and anxiety. Following data cleaning and reanalysis using the final dataset, prevalence was recalculated based on the standard cut-off of PHQ-9 ≥ 10 for moderate-to-severe depression and GAD-7 ≥ 10 for moderate-to-severe anxiety. This resulted in revised prevalence estimates of 22.9% for moderate-to-severe depression and 55.4% for moderate-to-severe anxiety.

### Pearson’s correlation between micronutrient intakes and depressive and anxiety symptoms among participants

Table 6 presents the correlation between selected micronutrient intakes and symptoms of anxiety and depression among adults living with disabilities. Overall, the findings show no statistically significant associations (p > 0.05) between any of the micronutrients assessed and mental health outcomes.

**Table 6:** Correlation between micronutrient intake and mental health symptoms among adults living with disabilities in Accra (*N* = 350)

Micronutrient	Anxiety (*r*, 95% CI, *p*-value)	Depression (*r*, 95% CI, *p*-vale)
Calcium (mg)	-0.040 (-0.142, 0.062) *p* = 0.453	-0.045 (-0.150, 0.057 *p* = 0.398
Iron (mg)	0.028 (-0.090, 0.146), *p* = 0.604	0.004 (-0.102, 0.109), *p* = 0.946
Zinc (mg)	-0.022 (-0.117, 0.074), *p* = 0.686	-0.059 (-0.163, 0.044), *p* = 0.268
Selenium (µg)	0.011 (-0.089, 0.111), *p* = 0.838	0.023 (-0.083, 0.129), *p* = 0.671
Thiamin (mg)	-0.002 (-0.106, 0.101), *p* = 0.966	0.018 (-0.088, 0.123), *p* = 0.743
Riboflavin (mg)	0.008 (-0.099, 0.115), *p* = 0.882	0.017 (-0.098, 0.132), *p* = 0.753
Niacin (mg)	0.008 (-0.096, 0.111), *p* = 0.888	0.084 (-0.010, 0.178), *p* = 0.118
Vitamin B6 (mg)	0.058 (-0.049, 0.165), *p* = 0.280	-0.036 (-0.138, 0.066), *p* = 0.499
Folate (µg)	-0.085 (-0.181, 0.012), *p* = 0.115	0.084 (-0.017, 0.185), *p* = 0.117
Vitamin B12 (mg)	-0.060 (-0.169, 0.050), *p* = 0.266	0.062 (-0.044, 0.167), *p* = 0.248
Vitamin C (mg)	-0.043 (-0.147, 0.060), *p* = 0.419	0.042 (-0.059, 0.142), *p* = 0.435
Vitamin A (µg RAE)	-0.001 (-0.095, 0.092), *p* = 0.993	-0.027 (-0.126, 0.073), *p* = 0.618
Vitamin D (µg)	-0.062 (-0.157, 0.033), *p* = 0.246	-0.079 (-0.178, 0.019), *p* = 0.139

*mg* milligram, *µg* microgram, *RAE* Retinol Activity Equivalent

*r* Pearson correlation coefficient

Correlation coefficients: 0.00-0.09 (negligible), 0.10–0.29 (weak), 0.30–0.49 (moderate), ≥ 0.50 (strong)

*CI* Confidence interval

*p*-value < 0.05 was considered statistically significant

The correlation coefficients for both anxiety and depression were generally weak and close to zero, indicating negligible relationships. For example, calcium intake showed a weak negative correlation with anxiety (*r* = -0.040, *p* = 0.453) and depression (*r* = -0.045, *p* = 0.398). Similarly, zinc (*r* = -0.022, *p* = 0.686) and vitamin C (*r* = -0.043, *p* = 0.419) demonstrated weak negative associations with anxiety, although these were not statistically significant.

Some nutrients exhibited weak positive correlations with mental health outcomes. For instance, niacin showed a weak positive correlation with depression (*r* = 0.084, *p* = 0.118), while vitamin B12 was weakly positively correlated with depression (*r* = 0.062, *p* = 0.248). However, these relationships were also not statistically significant.

Folate demonstrated a weak negative correlation with anxiety (*r* = -0.085, *p* = 0.115), and vitamin D showed a weak negative association with both anxiety (*r* = -0.062, *p* = 0.246) and depression (*r* = -0.079, *p* = 0.139). Despite these patterns, the wide confidence intervals crossing zero indicate uncertainty and lack of meaningful association.

In summary, the results suggest that micronutrient intake, as assessed in this study, was not significantly associated with symptoms of anxiety or depression in this population.

## Discussion

In this study of 350 adults living with disabilities in the Accra Metropolis, we identified a notable double burden of malnutrition: 28.8% of participants were underweight, while 31.4% were overweight or obese. At the same time, symptoms of common mental disorders were highly prevalent: 22.9% met criteria for moderate-to-severe depression on the PHQ-9, and 55.4% had moderate-to-severe anxiety on the GAD-7. These results point to substantial, co-occurring nutritional and mental health needs among adults with disabilities in this urban Ghanaian sample. Unexpectedly, micronutrient intakes, as assessed in this study, were not significantly associated with mental health symptoms in this population.

### Double burden of malnutrition

Our finding of coexisting underweight and overweight mirrors the urban nutrition transition documented across sub-Saharan Africa and Ghana specifically. National and regional reviews report rising urban overweight and obesity (with urban adult overweight/obesity estimates ranging widely but often between 25 and 45%), alongside persistent pockets of undernutrition in vulnerable groups [11–13]. For example, Ofori-Asenso and Agyeman [12] estimated the combined overweight/obesity prevalence of roughly 43% across Ghanaian adults in pooled analyses, with higher prevalence in urban populations. Our overweight/obesity figure (31.4%) is somewhat lower than pooled national urban estimates but remains substantial — and importantly, the simultaneous 28.8% underweight prevalence indicates intra-population heterogeneity that is consistent with reports of the double burden in urban LMIC settings [10, 11, 32].

Studies examining nutritional status specifically among people with disabilities are fewer but suggest elevated undernutrition risk among those with feeding difficulties, cerebral palsy, or severe disabilities, while those with mobility limitations or intellectual disabilities may have higher obesity risk — reflecting heterogeneous pathways to malnutrition in disability [7, 33–35]. For example, Jahan et al. [14] documented high undernutrition among children with cerebral palsy in Ghana, underscoring how disability type and functional limitation shape nutritional risk [33]. Our study adds to this literature by quantifying both underweight and overweight within an adult disability sample in an urban African context; the mixed pattern underscores the need for individualized assessment rather than group-level assumptions.

### Prevalence of depression and anxiety

The high prevalence of moderate-to-severe anxiety (55.4%) and depression (22.9%) symptoms in our sample exceeds typical community estimates for the general population in Ghana and many LMICs (where reported point prevalence for depressive symptoms often ranges lower, e.g., 10–20% depending on instrument and threshold) [36, 37]. However, our findings are consistent with recent reviews showing substantially elevated mental health burden among PLWD. A 2024 scoping review found consistently higher rates of depression among people with disabilities across settings, with multiple LMIC studies reporting prevalence well above 40% for depression in disability samples [20]. Country-level comparisons are complicated by differences in measurement thresholds, sampling frames, and access to care; nonetheless, the magnitude of psychological distress we observed aligns with international evidence that PLWD constitute a high-need group for mental health services [20, 21, 38].

### Nutrition–mental health intersection

Several plausible pathways link nutrition and mental health. Poor diet quality, micronutrient insufficiency (e.g., B vitamins, omega-3 fatty acids), and food insecurity have been associated with higher depressive symptoms in observational studies and some interventional trials (SMILES trial), though causality remains contested [24–26]. In disability populations, feeding difficulties, reduced dietary diversity, and social isolation may combine to increase both nutritional risk and psychological distress [7, 24, 27]. This study demonstrated that while macronutrient intake, particularly energy and protein, was relatively adequate, there was a high prevalence of micronutrient inadequacy, notably for iron, zinc, vitamin A, folate, and vitamin D. The overall Mean Adequacy Ratio (MAR) indicated moderate dietary quality, with only one-third of participants achieving adequate nutrient intake. These findings suggest a pattern of hidden hunger, where caloric sufficiency coexists with micronutrient deficiencies. The observed widespread inadequacy of iron, zinc, and vitamin A is consistent with evidence from similar low- and middle-income settings. National and regional studies consistently report that micronutrient deficiencies, particularly iron, zinc, and vitamin A, remain major public health concerns [39].

Although in this study, some micronutrients showed weak positive or negative trends with anxiety and depression symptoms, these associations were negligible and statistically non-significant, suggesting that micronutrient intake alone may not be a strong determinant of mental health outcomes in this population. The weak positive correlations may also reflect reverse causation, whereby individuals with more severe symptoms seek nutritional supplementation or dietary counseling. Alternatively, both high nutrient intake and mental health symptoms may be associated with healthcare-seeking behavior or socioeconomic status. Similar mixed findings have been reported in population-based studies, where both inverse and null associations between micronutrient intake and depressive or anxiety symptoms have been observed [24–26]. Longitudinal studies and intervention trials remain necessary to clarify causal pathways linking micronutrient status and mental health outcomes in this population.

### Potential explanations for our observed prevalences

Several contextual factors may explain our results. First, structural determinants such as poverty, limited social protection, and barriers to employment disproportionately affect PLWD in Ghana and worsen both food access and mental well-being [5, 6, 40]. Secondly, disability type and severity matter: individuals with physical or sensory impairments may have different barriers than those with intellectual or developmental disabilities; our stratified sample captured multiple disability types, which likely contributed to the mixed nutritional profile observed. Furthermore, urban living does not guarantee better nutrition or mental health; urban poverty and exposure to unhealthy processed foods can elevate obesity risk even as food insecurity persists [10, 11]. Finally, stigma, reduced social participation, and lack of disability-sensitive services amplify psychological distress [17, 18, 41].

### Measurement issues and methodological considerations in disability research

#### Anthropometry in disability populations

Standard BMI cut-offs are widely used but may misclassify adiposity in some people with disabilities, particularly those with altered body composition, contractures, or difficulty standing for height measurements [42–44]. We complemented BMI with bioelectrical impedance analysis (BIA), waist circumference, and waist-to-hip ratios where feasible; however, anthropometric interpretation in disability research requires caution. Literature reviews indicate BMI and waist circumference remain useful in many disability subgroups, but measurement validity varies, and alternative measures (e.g., BIA, tibia length estimation) are sometimes preferable [42, 43, 45].

### Mental health screening tools

The PHQ-9 and GAD-7 are validated screening instruments with substantial cross-cultural use, and prior Ghanaian studies report acceptable psychometrics in community samples [23, 31]. Nonetheless, screening tools identify probable cases and are not diagnostic; prevalence estimates may therefore overestimate clinical disorder if not confirmed by diagnostic interviews.

### Sampling and generalizability

Due to the absence of a complete sampling frame for adults with disabilities, we used stratified convenience recruitment; this approach allowed inclusion of diverse disability types but limits representativeness and generalizability beyond the study sample. Selection bias could inflate prevalence estimates if more symptomatic individuals were more likely to participate (care-seeking bias), although recruitment from multiple community and service locations aimed to reduce this risk.

### Cross-sectional design

Our design precludes causal inference. The observed associations (co-occurrence) could reflect reverse causation (e.g., depression leading to poor appetite and weight loss) or confounding by socioeconomic status, chronic comorbidity, or severity of disability. Future longitudinal or mixed-methods research is needed to disentangle pathways and identify modifiable targets.

### Implications for policy and practice

Our findings have several practical implications. First, disability-inclusive public-health programming should integrate routine nutrition screening (with disability-appropriate anthropometry) and mental-health screening in community disability services and primary care. Second, interventions must be tailored: undernutrition requires targeted feeding support and nutrient supplementation, whereas overweight/obesity prevention calls for access to healthy foods and adapted physical activity options. Third, social protection and livelihood programs for PLWD could reduce food insecurity and its downstream mental-health effects. Finally, research and monitoring systems should incorporate disability-disaggregated indicators into national nutrition and mental health surveys to drive policy action [1, 3, 7, 11].

### Future research directions

Longitudinal cohort studies and intervention trials are needed to test whether disability-sensitive nutrition interventions improve mental-health outcomes and functioning. Qualitative and mixed-methods work will also help elucidate lived experiences, barriers to healthy food access, and culturally appropriate strategies for psychosocial support among PLWD.

### Strengths and limitations

This study has several strengths. The use of validated screening tools and multiple nutritional assessment methods improved the reliability of the findings. The relatively large sample size (*n* = 350), inclusion of participants with different types of disabilities, and the assessment of both undernutrition and overnutrition also provide a broader picture of nutritional status in this population. However, some limitations should be noted. The cross-sectional design does not allow causal relationships to be established, and the use of convenience sampling may limit the generalizability of the results. While the sample size was deliberately oversampled beyond the calculated minimum, it may still be underpowered for subgroup analyses by disability type. The positive correlations observed between micronutrient intake and mental health symptoms should therefore be interpreted cautiously and require further investigation. In addition, the lack of adjustment for multiple comparisons and the inability to objectively verify dietary intake may have influenced the observed associations.

## Conclusion

Adults living with disabilities in urban Ghana face a high and co-occurring burden of malnutrition and common mental disorders, with over half experiencing moderate-to-severe anxiety (55.4%) and nearly a quarter reporting depression (22.9%). Addressing these intersecting challenges requires integrated, disability-inclusive nutrition, mental-health, and social-protection strategies within urban public-health systems. Further research is needed to understand the unexpected positive correlations between micronutrient intake and mental health symptoms. This study provides the first comprehensive assessment of nutritional status and mental health among adults with disabilities in urban Ghana.

## Data Availability

Available from the corresponding author upon reasonable request.
